# Designing a patient-centered care navigation intervention within a healthcare system for SUD care: a qualitative study

**DOI:** 10.1186/s13722-026-00712-z

**Published:** 2026-08-14

**Authors:** Mia A. Navarro, Theresa E. Matson, Hélène Chokron Garneau, Briana M. Patrick, Lia Chin-Purcell, Shubhi Sharma, Tyler D. Barrett, Rebecca C. Phillips, Joseph E. Glass

**Affiliations:** 1 Center for Dissemination and Implementation at Stanford, Department of Psychiatry and Behavioral Sciences, Stanford University School of Medicine, 1070 Arastradero Rd, 3rd Floor, CA Palo Alto, 94304 USA; 2 Kaiser Permanente Washington Health Research Institute, Seattle, WA USA; 3 Herbert Wertheim School of Public Health and Human Longevity Science, University of California, San Diego, CA USA; 4 Chestnut Health Systems, Lighthouse Institute, Eugene, OR USA

**Keywords:** Substance use disorder, Care navigation, Implementation, Barriers, Continuum of care, Qualitative

## Abstract

**Background:**

Healthcare systems often struggle to provide timely access to care for patients with substance use disorder, and many patients do not successfully engage with treatment. Care navigation interventions have emerged as a strategy to help patients navigate complex health systems, address barriers to care, and improve treatment initiation and engagement, but little information exists about how to optimize these interventions within health system settings. In preparation for the Addressing Barriers to Care for Substance Use Disorder Trial, a hybrid effectiveness-implementation study conducted in a large integrated healthcare system, we undertook a series of design activities including piloting, stakeholder engagement, and iterative process redesign. As part of this broader effort, this qualitative study elicited the perspectives of patients and clinicians on barriers and facilitators to substance use treatment and ways to optimize the implementation of care navigation within the healthcare system’s mental health access center.

**Methods:**

We conducted semi-structured interviews with 21 patients who sought treatment for substance use disorder and eight clinicians working in the mental health access center who assessed and referred patients to treatment. Interview transcripts were analyzed using the Rapid Group Analysis Process method, a novel rapid qualitative analysis method, to identify crosscutting themes related to care navigation.

**Results:**

Analyses identified several crosscutting themes that conveyed ways that care navigators could help connect patients to substance use treatment: by communicating effectively with patients, by leveraging substance use disorder resources, by supporting patient motivation, and by addressing logistical barriers to treatment. These themes closely aligned with the study’s existing care navigation protocol. Findings also informed several refinements to improve the fit of care navigation within the health system’s existing workflows.

**Conclusions:**

Identification of crosscutting themes via qualitative interviews confirmed the relevance of care navigation to the study population and helped optimize the intervention within the healthcare system. This study provides an example of how to incorporate stakeholder perspectives during intervention development and early implementation, particularly when adapting evidence-based approaches for new populations and settings.

**Trial registration:**

The clinical trial referenced in this study was prospectively registered at www.clinicaltrials.gov (NCT06729957) on December 9, 2024.

**Supplementary Information:**

The online version contains supplementary material available at https://doi.org/10.1186/s13722-026-00712-z.

## Introduction

United States healthcare systems must address the needs of patients seeking treatment for substance use disorders (SUD). Increased overdose death rates from 2019 to 2022 [1] revealed the challenges of delivering SUD care within healthcare systems. Patient needs along the continuum of care [2] are well-documented and of varying complexity to address. For example, patients may perceive that they could experience negative consequences from seeking treatment, face financial or transportation barriers that hinder treatment initiation, and experience dips in motivation or conflicting health needs that stall treatment engagement [3–10].

Care navigation has the potential to resolve complex needs among people with SUD. Care navigators work with patients to identify patient barriers to initiating treatment and then provide solutions from the community and healthcare system. [11] Care navigation has led to patient-centered treatment plans [3, 12] and directed financial support toward mitigating socioeconomic barriers to SUD care [3, 13–15] Care navigation has succeeded across various disease types (e.g., cancer, HIV, SUD) and settings (e.g., healthcare, jail, community) [16–22] SUD care navigation within the emergency department tripled outpatient SUD care engagement, compared to care as usual [23] Additional research is needed to understand how care navigation can be adapted and implemented across healthcare settings to support patients with SUD treatment needs.

Implementing and scaling SUD care navigation requires patient and clinician input. Prior care navigation studies for hospital, HIV, and end-of-life care obtained intervention feedback by interviewing patients [24–26] and interventionists [27–29] However, few studies delineated intervention and implementation determinants. Understanding these factors is essential for designing care navigation approaches that fit local healthcare contexts and can be sustained over time.

The Addressing Barriers to Care for Substance Use Disorder (ABC-SUD) study, a hybrid type I cluster-randomized effectiveness-implementation trial, exemplifies how to implement effective bridging interventions that link patients to SUD care. The study takes place in an integrated healthcare system’s mental health access center (MHAC), a triage center to assess patient needs and refer them to treatment. The ABC-SUD study aims to develop and implement a care navigation intervention that helps patients referred to addiction treatment successfully engage in treatment. The ABC-SUD study also seeks to provide an implementation science lens for care navigation, creating generalizable knowledge that guides future implementers of care navigation within different contexts. This study was a research project of the National Institute on Drug Abuse Center of Excellence in Implementation Science (P50DA054072), involving researchers from multiple institutions. Details can be found in the protocol paper [30].

To contribute to care navigation research landscape, [24, 31, 32] this qualitative study was conducted to inform the design of a care navigation intervention, including how to best address patient needs and barriers to treatment. We also sought to understand how the care navigation intervention could be optimized for implementation within the health system. We conducted semi-structured interviews with patients and clinicians from MHAC to capture their perspectives and recommendations for how to improve the initial study protocol.

## Methods

### Study design

Reporting was informed by the COnsolidated criteria for REporting Qualitative (COREQ) research checklist [33] We conducted one-time, semi-structured patient interviews between October 2023 and July 2024 and clinician interviews between October 2024 and February 2025. This qualitative study was approved by the Kaiser Permanente Interregional Institutional Review Board, which granted ethical approvals including waivers of written consent and Health Insurance Portability and Accountability Act authorization.

### Setting

Kaiser Permanente Washington (KPWA) is a large integrated healthcare and insurance coverage system in Washington State that provides health and preventive services to over 700,000 people. KPWA’s integrated group practice includes 33 facilities (primary care clinics, specialty centers, urgent care), and KPWA’s contracted care network provides most of its specialty addictions, emergency department, and hospital care. KPWA serves patients covered by commercial insurance, self-pay, Medicare Advantage, and Medicaid.

Patients seeking substance use and mental health care are connected to MHAC, a triage service, either by self-referral or a referral from a KPWA clinician. During this first call with MHAC clinicians, patients complete a 30–45 min telephonic or video-based mental health assessment in which clinicians gather information about mental health, substance use, and SUD symptoms, elicit patient goals for treatment, determine what resources are available and accessible (e.g., factoring in insurance coverage and travel) and create a personalized treatment plan. Treatment plans may include care that is provided inside of KPWA, such as outpatient group-based treatment and medication management from KPWA’s addiction recovery service (2021–2024), counseling from the mental health and wellness department, or medications from psychiatry and primary care. They may also refer patients to resources outside of KPWA when internal treatment is unavailable or does not meet all patient needs (e.g., higher-level of care needed). These referrals are made to community agencies that accept KPWA insurance, many of which provide outpatient, intensive outpatient, or residential specialty addiction treatment. Additional details about the assessments and the health system are available in the published study protocol [30].

### Eligibility and recruitment

#### Patients

Eligible patients were (1) ages 18 or older upon recruitment; (2) fluent in English or Spanish; and (3) contacted or referred to MHAC to seek SUD treatment. Potentially eligible patients were identified by a programmer on the research team using electronic health records and were then sent a recruitment letter by the research institute’s survey research program (SRP). The programmer oversampled demographic, socioeconomic, and language subgroups to promote adequate representation, including those who had received medical financial assistance, people with Medicaid insurance, American Indian or Alaska Native, Asian, Black, Hispanic, Native Hawaiian or Pacific Islander, and Spanish-speaking patients [34] The recruitment letter included a two-dollar pre-incentive and an information sheet with study details and contact information. SRP followed up by phone and conducted an eligibility screening to confirm patients were seeking SUD care, specifically, in the past 6 months. The SRP conducted an informed consent process with patients who agreed to an interview.

The SRP attempted to contact all 68 patients who received a recruitment letter. Two patients were deemed ineligible because they did not seek SUD treatment in the past six months. Thirty-two patients were never reached over the phone within the maximum of three phone calls or provided invalid phone numbers. Among the 36 patients contacted, 13 patients declined, mostly due to being busy. Twenty-one patients provided verbal consent and were interviewed.

#### Clinicians

Eligible clinicians were licensed, Masters-level mental health clinicians (e.g., Licensed Mental Health Counselors, Licensed Marriage and Family Therapists, and Licensed Clinical Social Workers) in MHAC. Department managers provided a list of clinicians to the research team and emailed all clinicians to introduce the study and inform them that SRP staff would send a recruitment email inviting them to join the study. Of the 33 clinicians contacted by SRP via email, nine responded. One clinician then declined due to scheduling constraints, and eight clinicians provided verbal consent and were interviewed. All clinicians on the list provided by department managers were also invited to participate in the trial.

### Interviews

Patient interviews were conducted between October 23, 2023, and July 9, 2024, concluding on the first day of the pilot phase (July 9, 2024 – November 27, 2024). Clinician interviews were conducted between October 4, 2024, and February 13, 2025, spanning the pilot phase and the first two months of the 14-month trial (December 16, 2024 – July 17, 2026).

#### Survey research program

SRP conducted semi-structured interviews with patients and clinicians. Two of the patient interviews were conducted in Spanish. Interviewers were professional, experienced staff that held bachelor’s or master’s degrees and received foundational training in participant recruitment and professional development of interviewer skills and several hours of study-specific training.

#### Interview domains

Patients and clinicians were asked to identify barriers and facilitators to SUD care and mechanisms by which care navigation can improve SUD care. Patients received additional questions regarding experiences seeking SUD care, support they would like from care navigators, and preferred communication methods with care navigators. Clinicians received additional questions about how best to integrate care navigation into KPWA, how care navigation can benefit clinicians, resources that care navigators should leverage, barriers and facilitators of implementation within KPWA, concerns with the ABC-SUD protocol, and their expected outcomes after participating in the ABC-SUD study. Interview guides were pilot-tested among the study team (Appendices C-D).

### Data collection

Interviewers used Microsoft Teams to conduct 45- to 60-minute audio-only interviews and recorded audio for transcription. Recordings were professionally transcribed and translated to English, if needed. Identifiers were removed from transcripts to maintain confidentiality. De-identified transcripts were uploaded to a secure enterprise cloud-based data management platform for cross-institute collaboration. Interview participants received gift cards and thank you letters.

### Data analysis

The research team performed the Rapid Group Analysis Process (Rap-GAP), a novel rapid qualitative analysis method [35] The deductive Rap-GAP method allowed quick translation of findings into ABC-SUD protocol, was an approachable method, and ensured the research team deduced all critical findings.

The Rap-GAP method comprises five steps to plan and conduct the analysis. Rap-GAP leads (MN, TM, JG) first created a Microsoft Excel form organized by interview domain in a similar manner to the interview guide. Leads provided team member analysts, who all had prior qualitative experience (BP, LCP, SS), with Rap-GAP training and assigned transcripts to each analyst. Analysts compiled important notes and insights within each domain on separate Excel files. Notes and insights were exported to Miro, a virtual whiteboard workspace, to visually represent the compiled themes (Appendix Figure A.1). Rap-GAP leads, data extractors, and two mental health clinicians who served as care navigators on this study participated in the Group Analysis Process by collaboratively grouping insights into themes over a series of meetings. Findings were then discussed with a project collaborator (HCG) for feedback. Virtual whiteboards were edited, labeled, and annotated to reflect consensus on subthemes and themes, which were exported to an aggregated Excel file. Interview participant feedback on the Rap-GAP findings was not sought. Relevant insights and illustrative quotes were shared with health system clinicians during training and with department leaders in an executive summary to seek feedback. Fifth, Rap-GAP leads identified the most salient findings and linked these findings with supportive quotes. Additional details about the Rap-GAP method can be found in prior literature [35] and in Appendix (Appendix Figure A).

## Results

The following sections report on participant characteristics, present qualitative findings related to patient experiences seeking care, and describe four crosscutting themes related to care navigation and implementation within an existing healthcare system. Themes are then assessed alongside the ABC-SUD care navigation protocol to illustrate how findings were translated into protocol elements.

### Participant demographics

The modal age of patients was between 40 and 59 years (*n* = 10, 47.6%; Table 1). Most patients self-identified as White (*n* = 7, 33.3%) or Hispanic (*n* = 7, 33.3%) (see Table 1). Most clinicians were female (*n* = 6, 75%) and self-identified as White (*n* = 6, 75%), similar to the KPWA clinician population.

**Table 1:** Characteristics of qualitative interview participants

Characteristic*	Patients (*N* = 21)	Clinicians (*N* = 8)
Age		
18–39	8 (38.1)	2 (25.0)
40–59	10 (47.6)	5 (62.5)
≥60	3 (14.3)	1 (12.5)
Sex		
Female	10 (47.6)	6 (75.0)
Male	11 (52.4)	2 (25.0)
Race and ethnicity		
Asian	2 (9.5)	1 (12.5)
Black	2 (9.5)	1 (12.5)
Hispanic	7 (33.3)	0 (0.0)
White	7 (33.3)	6 (75.0)
Multiracial	1 (4.8)	0 (0.0)
Other/Unknown	2 (9.5)	0 (0.0)

* Values are represented as n (%)

### Patient experiences seeking SUD care

Patients experienced many pathways into SUD care. Patients often first engaged with MHAC after interacting with health system staff (e.g., physicians), seeking treatment for themselves, or after a recommendation to seek treatment by external counseling or social service agencies.

Overall, patients who interacted with MHAC reported positive experiences seeking SUD care (Table 2 Q1). Patients appreciated being provided multiple treatment options and treatment plans that addressed co-occurring mental health disorders. Patients reported difficulties during the follow-up between initial contact and linkage to care with one patient wanting more direct guidance about how to seek treatment and more information “about which path [the clinician] thought [they] should take” (PT #33). Some patients reported less supportive interactions when seeking care outside the context of an MHAC visit. One patient was given a list of agencies to navigate on their own, while others were given only one option that did not align with their treatment goals or preferences (e.g., residential SUD treatment).

**Table 2:** Key insights and exemplar quotes from qualitative interviews with patients and clinicians

Key insight	Quote ID	Quote
Patient experiences with SUD care	Q1	“[The clinician] helped me - I felt that they were very effective at listening, they asked appropriate questions, and I felt that at the end they presented options and suggested the course that I ended up taking. I didn’t feel coerced in any way. It was like you could do this, do this, you may want to consider this in making the decision. Yeah, it was very helpful, they were really great.” (PT #55)
Patients desire effective communication during the treatment seeking process.	Q2	“I think multiple avenues to reach out to the patient, meaning some are going to respond best to phone calls. Some would respond best to emails, some would respond best to secure messages in their Kaiser inbox and I don’t know if multiple pathways to reach out to them is part of [care navigation] but I think it could be helpful if it is.” (MHCC #29)
Patients desire support leveraging existing SUD resources.	Q3	“A lot of people aren’t interested in the group piece. I would say there [are] a lot of individuals who are explaining, contemplative still, whether they want to stop use or not stop use they’re nervous that the response is going to be you have to completely stop all use period. And I have not once heard anything about harm reduction options… It’s scary for people who are actively using to think that their expectation is going to be you have to stop using period… It’s a lot more individualized and a lot less shame, a lot less pressure and expectation.” (MHCC #25)
Patients desire support to improve their motivation to seek SUD care.	Q4	“[Hand holding] will engage [patients] in treatment and support services. Or two, they’ll decide they’re not going to engage in treatment. And for those patients you’re still having a relationship with them so that you’re still making sure they maybe have other services that could be provided. Um, and they’re not just kind of hiding again in shame or embarrassment about not following through. That the door is open and they’re witnessing us be supportive of their decision on what to do… So if we can suggest something else, if they don’t follow our recommendations then we support them in something else, I think that’s supportive,” (MHCC #8)
Patients desire assistance addressing logistical barriers to treatment.	Q5	“…I called customer service and they sent me an email with a list but that was just out of date… so I actually had to go to the emergency room… [the nurse] came back with just another list, and that list was up to date. So I called all the numbers on there and finally got into one.” (PT #13)

*MHCC = mental health care coordinator; PT = patient; SUD = substance use disorder.* Quotes were extracted from 21 patient transcripts and organized by crosscutting themes revealed in the Rapid Group Analysis Process (Rap-GAP). Quote IDs are referenced in-line in the text to support relevant statements

### Synthesis of crosscutting themes

Table 2 compiles crosscutting themes of care navigation and exemplar quotes from the Rap-GAP. Additional subthemes were organized using the socioecological model within interview domains (Appendix Figure B.1 and Table B.1).

#### Communicating effectively with care navigators and patients

Patients shared ideas about how care navigators could help patients better understand the treatment seeking process, including several recommendations for effective communication. Several patients recommended that care navigators use modern methods to communicate with patients throughout their process of initiating care. One patient wished “there was something in place where [they] could have gotten text notifications, something more pushing …” (PT #51). This aligns with patients’ preference for text communication with care navigators upon initial contact and over time. Though many patients preferred phone calls when communicating with care navigators upon first contact and throughout the intervention. One clinician recommended that care navigators use multiple communication methods to contact patients to improve linkage to SUD care (Table 2 Q2).

Patients also emphasized the importance of “soft skills” during communications, regardless of the method. Both patients and clinicians emphasized that care navigators should be empathic and demonstrate good listening skills while compassionately guiding patients to SUD care. Clinicians observed that a fear of being judged about substance use prevents patients from speaking candidly, making it difficult to develop rapport with patients.

Timely outreach by care navigators was viewed as critical. Several patients wanted initial contact with care navigators to occur within a few days of their initial clinician assessment and contact thereafter to occur weekly or as needed by the patient. In summary, care navigation could potentially improve SUD care by improving timing, quality, and frequency of communication between patients and care teams (Appendix Table B.2).

#### Leveraging resources for SUD care

Patients and clinicians, “think it would be helpful for the navigator… to have resources [and] to share those resources [with patients] even before treatment,” (MHCC #30). Clinicians suggested that being able to offer a variety of treatment options (e.g., abstinence vs. harm reduction, group vs. individual format; Table 2 Q3) and basic resources (e.g., transportation) could help improve receptivity to treatment and/or facilitate linkage to SUD treatment. This aligns with clinician requests for psychoeducational SUD resources and multiple treatment options, transportation to treatment centers, language interpreters, and financial resources (e.g., money to cover cost-of-living and medical expenses, health insurance coverage) from care navigators.

Patients appreciated services offered within the healthcare system such as KPWA mental health resources, including individual counseling and psychiatry, KPWA wellness coaches, and KPWA bereavement services. However, clinicians noted gaps in these services. Specifically, clinicians noted that the lack of an intensive outpatient treatment program within KPWA may make it harder to navigate patients needing that level of care. Extended inpatient treatment is sometimes the only available alternative for patients needing a higher level of care, posing a barrier to those with strict work obligations. Clinicians also stated the supply does not meet patient demand and that they “need to have other ideas of what we do with patients when they can’t get in right away” (MHCC #8).

Patients stated that receiving resources and curated treatment options to match their preferences are critical to improving the patient experience and quality of SUD care. Ensuring care navigators have access to up-to-date information on available resources facilitates care navigation implementation.

#### Supporting patient motivation to seek SUD care

Patients appreciated encouragement from their support system in a non-stigmatizing manner. Support networks can comprise a range of individuals, such as family members, friends, professionals, and peers with lived SUD experience. One patient reflected on the value of this support, “I didn’t feel like I’m a failure, like judgment of character, more a disease kind of thing and it’s ok to ask for help” (PT #1).

Clinicians hoped care navigation would support patients throughout the continuum of care and address personal stigma, which can improve sentiments about SUD care and engaging with care navigation (Table 2 Q4). To this end, care navigators can employ motivational interview techniques that elicit patients’ own motivations for change, build rapport through good communication (see Sect. "Communicating effectively with care navigators and patients") thereby bolstering their support system, and help patients stay accountable to themselves through summaries of next steps and consistent follow-up.

#### Addressing logistical barriers to SUD treatment

Several aspects of navigating the continuum of care can delay SUD treatment initiation. Patients were referred to distant treatment centers with, “[long drives that] really didn’t appeal to [them],” (PT #33). Patients faced several obstacles to scheduling appointments for themselves, such as having to decipher treatment eligibility criteria (i.e., accepted insurance carriers). One clinician recounted that their patients have had to turn to emergency resources, which, “may not be necessary if they can go into a detox facility right away, and they’re assessed as meeting criteria for that” (MHCC #8). Other patients were left on their own to navigate outdated resources to find eligibility criteria or to ensure treatment was covered by insurance (Table 2 Q5).

Participants stated that care navigation can improve care by providing logistical support. Patients specifically noted that if care navigators called treatment centers on their behalf to determine eligibility and schedule appointments, this would, “cut out the middlemen and speed up the process,” (PT #13). Care navigators specifically can, “expedite the process, just to reduce the wait time and the need to talk to other people,” (PT #13).

Patients and clinicians would value care navigators that can problem-solve patients’ issues, as clinicians noted it is difficult to navigate the continuum of care while still using substances. Moreover, patients found it important that care navigators “actually do get back to you.” (PT #55) and are, “willing to want to help,” (PT #60). Achieving such goals requires care teams to effectively manage caseloads and to coordinate efforts.

### Evaluation of crosscutting themes in comparison to the existing ABC-SUD protocol

Table 3 outlines how existing ABC-SUD care navigation intervention protocol items relate to the four crosscutting themes identified in this study. The care navigation protocol specifies the broad intervention goals of care navigation (i.e., core functions) along with a variety of intervention activities (i.e., forms) that can be used to accomplish those core functions [30, 36, 37] Three qualitative themes were directly addressed in intervention activities: leveraging SUD resources, supporting patient motivation, and/or addressing logistical barriers (Table 3). For instance, one can improve patient motivation with the intervention activity, “praise change efforts,”. The protocol also addressed the theme of communicating effectively with patients. For instance, the protocol guides care navigators to explain care navigation goals on first contact, to assess and use the preferred contact method, and requires timely contact with referred patients. It also provides guidelines about communication frequency.

**Table 3:** Incorporation of crosscutting themes and related feedback into ABC-SUD protocol

Theme	Summary of Existing ABC-SUD Protocol	Interviewee Feedback and Recommended/Executed Protocol and Implementation Changes
Communicating effectively with patients	Care navigators reach out to patients via phone and/or secure message within one week (ideally next day). Care navigators explain the study objective upon first contact. The care navigator continues communication with the patient via phone and patient portal messaging as needed (exact cadence driven by patient availability and preference) until the patient and care navigator mutually agree on an end date (typically once the patient has initiated and engaged with treatment).	**Feedback**: Patients prefer text messaging and clinicians recommend offering multiple communication modes and note that phone calls may miss nuances expressed by patients. **Recommendations**: Utilize smartphone text-based messaging for care navigation communications, allowing patients to conveniently use existing phone functionality. Incorporate online meeting software (e.g., Zoom) for care navigation sessions in addition to phone calls to capture more communication nuances. **Executed Changes**: Frequency of communication was driven by patient preferences, and at minimum, care navigators would reach out weekly. Within the intervention team, we reinforced our goals to contact patients quickly after expressing interest in care navigation. Relatedly, we set up new, streamlined communication workflows between care navigators and clinicians via Microsoft Teams to speed up communication regarding patient cases.
Leveraging SUD resources	Care navigators address resource constraints through the following process: ● assess barriers to care (e.g., ask open-ended questions, administer barriers checklists); ● problem-solve ways to overcome barriers; ● offer resources that address barriers (e.g., non-emergency medical transportation services); ● assist with linkage to treatment (e.g., finding agencies, handling administrative issues); and ● facilitate communication with health care systems (e.g., clinicians, resource specialists, treatment agencies)	**Feedback**: Patients would like to see available resources before discussing a treatment plan. Also, patients and clinicians requested care navigators have up-to-date information about resources. **Recommendation**: Establish bi-directional communication and information-exchange channels between referring clinicians and care navigators to share information about resources and suitability of patient treatment plans **Executed Changes**: We arranged additional education for clinicians in the form of a brief seminar about SUD diagnosis and SUD treatment needs. Enhanced documentation tools supporting SUD assessment and treatment planning.
Supporting patient motivation	Care navigators address patient motivation and social support for SUD treatment via the following: ● build supportive relationship with patient; ● explore concerns about substance use, readiness to change, and optimism about success; ● praise change efforts, affirm client’s ability to reduce substance use and seek care; ● maintain regular contact while patient seeks, initiates, and engages in treatment; ● facilitate communication with health care systems (e.g., clinicians, resource specialists, treatment agencies); ● seek to understand and, if needed, improve patient’s relationship with health care systems (e.g., collect questions, educate); and ● accommodate patient preferences.	**Feedback**: Patients appreciate patient-centered support from care navigators and support systems and interactions that appear free of judgement. **Recommendations**: Findings reinforce the importance of maintaining supportive relationships, regular follow-up, and flexibility in the duration of navigation support for patients. **Executed Changes**: Added a section to the clinician trainings on using patient-centered language. We provided scripts for offering care navigation services using neutral language, including language that is free of stigmatized terms.
Addressing logistical barriers to treatment	Clinicians place a referral for care navigation via the electronic health record. Study team co-designs electronic health record tools and workflows that work best for clinicians and patients. Care navigators address logistical barriers by doing the following: ● problem-solve ways to overcome barriers; ● maintain regular contact while patient seeks, initiates, and engages in treatment; ● assist with linkage to treatment (e.g., finding agencies, handling administrative issues); ● facilitate communication with health care systems (e.g., clinicians, resource specialists, treatment agencies); and set up workflows ● seek to understand and, if needed, improve patient’s relationship with health care systems (e.g., collect questions, educate).	**Feedback**: Patients would appreciate referrals to more local treatment centers. Patients would also appreciate support ensuring they meet eligibility criteria at treatment centers and have eligible insurance coverage. **Recommendations**: Engage clinicians to encourage them in making high-quality and low-barrier treatment plans. Educate patients about treatment options, clarifying treatment plans with referring clinicians when needed. Help prepare patients to self-advocate when seeking treatment. Warmly hand off patients to treatment centers. If more support is needed, make the call to treatment centers with patients on the line. **Executed Changes**: Few additional changes needed; we extended navigation services for patients requiring continued support after medically managed detoxification to ensure barriers could be addressed longitudinally while they progress through the continuum of care.

*SUD = substance use disorder. *Feedback obtained during interviews is translated into actionable recommendations to optimize the existing protocol. Intervention forms are activities that can be undertaken to achieve a particular core function of care navigation. Prescribing intervention forms provides direction on how to administer the intervention, while specifying core functions allows for intervention flexibility while maintaining fidelity to the intervention’s goals

Although the majority of patient and clinician recommendations aligned with existing ABC-SUD protocol functions and intervention forms, qualitative findings informed several recommendations, several of which were executed during the trial. Executed changes included improvements to communication workflows between care navigators and clinicians (e.g., real-time, bidirectional messaging), improving ease of use for multiple communication modalities available to patients, reinforcement of rapid outreach and follow-up procedures, extension of navigation services for certain patients requiring continued support after medically managed detoxification, additional clinician education related to SUD treatment, clinician engagement strategies to promote adoption (e.g., recognizing successes), and enhancements to documentation tools supporting SUD assessment and treatment planning. (Table 3)

## Discussion

Care navigation has an adaptable framework for many healthcare contexts and conditions. In the case of SUD, it can provide parameters for ensuring a smooth linkage to SUD treatment. This qualitative study was used to reveal and address barriers experienced by patients to better tailor care navigation to the target patient population and health system for future implementation efforts.

Our findings align with prior research describing effective communication between care navigators, clinicians, and patients as inherent to care navigation. Scoping reviews described electronic health record (EHR) tools as a way to facilitate effective communication between clinical team members [38, 39] The ABC-SUD protocol integrates EHR tools, such as communication templates and text shortcuts (“smart phrases”), that ease communication between care navigators and clinicians to improve patient care. Interview participants in the current study further emphasized the need to enhance communication procedures within existing workflows to align with patient preferences. An example of this is that patients recommended smartphone text-based messaging for care navigation communications and online meeting software (e.g., Zoom) for care navigation sessions.

Problem-solving barriers to SUD treatment via care navigation can improve linkage to SUD treatment. Problem solving has also been identified as a mechanism of care navigation for dementia treatment [40, 41] Key methods to address logistical barriers that aligned with patients’ barriers to treatment, such as transportation services and facilitating childcare responsibilities, have been discussed in the literature [42] We expanded on this prior work and exemplified how to adapt intervention and implementation activities for SUD care within a large healthcare system.

Our analysis also identified supporting patient motivation and leveraging SUD resources as another priority of care navigation. Prior research discussed motivational barriers at the individual level and identified supportive relationships with clinicians as a way to overcome such barriers [43,44] Beyond building patient support networks, prior research also noted drug use program participation is catalyzed by community support and trust [44] Though Washington state has been deemed a resource-abundant state in the US, [45] the ABC-SUD study team and other implementation researchers must consider how best to support patients and clinicians when demand outpaces or misaligns with resource availability.

Patient and clinician feedback is critical to implement and scale care navigation to new settings. Prior care navigation studies have interviewed patients [24–26] and interventionists [27–29] to gain intervention feedback. Studies varied in their purpose and timing, with some interviews confirming protocols post hoc or informing future care navigation interventions. In the current study, the timely application of a rapid qualitative group analysis approach allowed us to use interview results to tailor a fully drafted care navigation interview protocol while it was still being used in a care setting. Traditional qualitative analytic approaches are much slower and interview timing decisions can limit the ability to obtain real-time feedback. Though studies that conduct qualitative interviews after an intervention concludes can tell a full story about intervention implementation and provide insight into decisions that lead to sustainment or non-sustainment, they are unable to address identified barriers in real time. This current study prioritized collecting quality improvement feedback in the design and early implementation phase in hopes to accelerate knowledge sharing about effective care navigation interventions.

This study exemplifies a co-designed care navigation intervention via patient and clinician interviews by mapping themes onto protocol items. Crosscutting themes were addressed in the protocol through recommended intervention activities which care navigators can leverage during patient interactions. The study team adopted the Core Functions and Forms paradigm so care navigators can tailor the intervention to participants (i.e., forms), while also maintaining fidelity to the intervention’s objectives (i.e., functions) [36,37] We hope highlighting the recommended forms promotes transparency of implementation and informs future implementers how to achieve intervention functions.

Our study had limitations. First, as a qualitative study with a goal of understanding needs of a specific context, results are not intended to generalize across settings or people with SUD. However, we prioritized understanding a variety of voices by sampling patients and clinicians, and purposely sampling Spanish speakers, in hopes to tailor the intervention to multiple perspectives within this health system context. Second, transcripts were assigned to three analysts, who may have differed in data interpretation. However, this was by design of the Rap-GAP method that prioritizes tapping into the unique perspectives of a variety of individuals who hold different expertise and timeliness of translating findings into practice. Third, some participants struggled to distinguish the intervention (care navigation) from its implementation process, making it difficult to generate tailored insights within each domain. Future work should explore ways to optimize methods for obtaining implementation-related feedback from collaborators who are often indirectly involved in implementation decision-making.

This qualitative study of patients and clinicians identified several critical factors related to successfully designing and implementing care navigation: effective communication between care teams and patients, addressing patient motivation and logistical barriers along the continuum of care, and facilitating patient access to resources. It provides an example of how to incorporate stakeholder perspectives during intervention development and early implementation, particularly when adapting evidence-based approaches for new populations and settings. Future research should evaluate whether tailored adaptations improve treatment linkage, patient engagement, and implementation outcomes as care navigation interventions are scaled and sustained across healthcare systems.

## Supplementary Information

Below is the link to the electronic supplementary material.

Supplementary Material 1

Supplementary Material 2

Supplementary Material 3

Supplementary Material 4

## Data Availability

Due to the sensitive nature of the questions asked in this study, interview participants were assured raw data would not be shared publicly.

## References

[1] Garnett MF, Miniño AM. Drug overdose deaths in the United States 2003–2023. 2024. Accessed October 23. 2025. 10.15620/cdc/170565.

[2] Evashwick C. Creating the continuum of care. Health Matrix. 1989;7(1):30–9. 10293297

[3] Krawczyk N, Rivera BD, Chang JE, et al. Strategies to support substance use disorder care transitions from acute-care to community-based settings: a scoping review and typology. Addict Sci Clin Pract. 2023;18(1):67. 10.1186/s13722-023-00422-w. PMC10621088 37919755

[4] Beieler AM, Klein JW, Bhatraju E, Iles-Shih M, Enzian L, Dhanireddy S. Evaluation of Bundled Interventions for Patients With Opioid Use Disorder Experiencing Homelessness Receiving Extended Antibiotics for Severe Infection. Open Forum Infect Dis. 2021;8(6):ofab285. 10.1093/ofid/ofab285. PMC8231362 34189180

[5] Dunkley CA, Carpenter JE, Murray BP, et al. Retrospective Review of a Novel Approach to Buprenorphine Induction in the Emergency Department. J Emerg Med. 2019;57(2):181–6. 10.1016/j.jemermed.2019.03.029. 31060846

[6] Englander H, Dobbertin K, Lind BK, et al. Inpatient Addiction Medicine Consultation and Post-Hospital Substance Use Disorder Treatment Engagement: a Propensity-Matched Analysis. J Gen Intern Med. 2019;34(12):2796–803. 10.1007/s11606-019-05251-9. PMC6854181 31410816

[7] O’Connell MJ, Flanagan EH, Delphin-Rittmon ME, Davidson L. Enhancing outcomes for persons with co-occurring disorders through skills training and peer recovery support. J Ment Health. 2020;29(1):6–11. 10.1080/09638237.2017.1294733. 28282996

[8] Rochat S, Wietlisbach V, Burnand B, Landry U, Yersin B. Success of referral for alcohol dependent patients from a general hospital: predictive value of patient and process characteristics. Subst Abus. 2004;25(1):9–15. 10.1300/J465v25n01_03. 15201107

[9] Sorensen JL, Masson CL, Delucchi K, et al. Randomized trial of drug abuse treatment-linkage strategies. J Consult Clin Psychol. 2005;73(6):1026–35. 10.1037/0022-006X.73.6.1026. 16392976

[10] Wakeman SE, Kane M, Powell E, et al. A hospital-wide initiative to redesign substance use disorder care: Impact on pharmacotherapy initiation. Subst Abus. 2021;42(4):767–74. 10.1080/08897077.2020.1846664. 33270549

[11] McBrien KA, Ivers N, Barnieh L, et al. Patient navigators for people with chronic disease: A systematic review. PLoS ONE. 2018;13(2):e0191980. 10.1371/journal.pone.0191980. PMC5819768 29462179

[12] Regan S, Howard S, Powell E, et al. Emergency Department-initiated Buprenorphine and Referral to Follow-up Addiction Care: A Program Description. J Addict Med. 2022;16(2):216–22. 10.1097/ADM.0000000000000875. 34145185

[13] Edwards FJ, Wicelinski R, Gallagher N, McKinzie A, White R, Domingos A. Treating Opioid Withdrawal With Buprenorphine in a Community Hospital Emergency Department: An Outreach Program. Ann Emerg Med. 2020;75(1):49–56. 10.1016/j.annemergmed.2019.08.420. 31732373

[14] Gryczynski J, Nordeck CD, Welsh C, Mitchell SG, O’Grady KE, Schwartz RP. Preventing Hospital Readmission for Patients With Comorbid Substance Use Disorder. Ann Intern Med. 2021;174(7):899–909. 10.7326/M20-5475. PMC13412153 33819055

[15] Mitchell SG, Monico LB, Stitzer M, et al. How patient navigators view the use of financial incentives to influence study involvement, substance use, and HIV treatment. J Subst Abuse Treat. 2018;94:18–23. 10.1016/j.jsat.2018.07.009. PMC6404543 30243412

[16] Creasy SL, Sweet S, Myers JJ, et al. eHealth-Enhanced Peer Navigation for Substance Use Treatment and HIV Prevention Service Linkage for Young Adults Surveilled by the Criminal Legal System: Protocol for a Pilot Randomized Trial Study. JMIR Res Protoc. 2024;13:e54815. 10.2196/54815. PMC11005443 38530335

[17] Freund KM, Battaglia TA, Calhoun E, et al. Impact of Patient Navigation on Timely Cancer Care: The Patient Navigation Research Program. JNCI: J Natl Cancer Inst. 2014;106(6):dju115. 10.1093/jnci/dju115. PMC4072900 24938303

[18] Freund KM, Haas JS, Lemon SC, et al. Standardized activities for lay patient navigators in breast cancer care: Recommendations from a citywide implementation study. Cancer. 2019;125(24):4532–40. 10.1002/cncr.32432. PMC6891116 31449680

[19] Karver TS, Barrington C, Donastorg Y, et al. Exploring peer navigation and support in the quality of HIV care experiences of female sex workers in the Dominican Republic. BMC Health Serv Res. 2022;22:56. 10.1186/s12913-021-07439-4. PMC8753897 35016659

[20] Lasser KE, Murillo J, Lisboa S, et al. Colorectal Cancer Screening Among Ethnically Diverse, Low-Income Patients: A Randomized Controlled Trial. Arch Intern Med. 2011;171(10):906–12. 10.1001/archinternmed.2011.201. 21606094

[21] Metsch LR, Feaster DJ, Gooden L, et al. Effect of Patient Navigation With or Without Financial Incentives on Viral Suppression Among Hospitalized Patients With HIV Infection and Substance Use. JAMA. 2016;316(2):156–70. 10.1001/jama.2016.8914. PMC5339876 27404184

[22] Ramirez A, Perez-Stable E, Penedo F, et al. Reducing time-to-treatment in underserved Latinas with breast cancer: The Six Cities Study. Cancer. 2014;120(5):752–60. 10.1002/cncr.28450. PMC3949173 24222098

[23] Anderson ES, Rusoja E, Luftig J, et al. Effectiveness of Substance Use Navigation for Emergency Department Patients With Substance Use Disorders: An Implementation Study. Ann Emerg Med. 2023;81(3):297–308. 10.1016/j.annemergmed.2022.09.025. 36402631

[24] Kokorelias KM, Gould S, Das Gupta T, Ziegler N, Cass D, Hitzig SL. Implementing patient navigator programmes within a hospital setting in Toronto, Canada: A qualitative interview study. J Health Serv Res Policy. 2022;27(4):313–20. 10.1177/13558196221103662. 35593462

[25] Rocque GB, Dionne-Odom JN, Sylvia Huang CH, et al. Implementation and Impact of Patient Lay Navigator-Led Advance Care Planning Conversations. J Pain Symptom Manag. 2017;53(4):682–92. 10.1016/j.jpainsymman.2016.11.012. PMC6559345 28062341

[26] Sullivan KA, Schultz K, Ramaiya M, Berger M, Parnell H, Quinlivan EB. Experiences of Women of Color with a Nurse Patient Navigation Program for Linkage and Engagement in HIV Care. AIDS Patient Care STDs. 2015;29(S1):S49–54. 10.1089/apc.2014.0279. PMC4283065 25457920

[27] Gunn CM, Clark JA, Battaglia TA, Freund KM, Parker VA. An Assessment of Patient Navigator Activities in Breast Cancer Patient Navigation Programs Using a Nine-Principle Framework. Health Serv Res. 2014;49(5):1555–77. 10.1111/1475-6773.12184. PMC4213049 24820445

[28] Jean-Pierre P, Hendren S, Fiscella K, et al. Understanding the Processes of Patient Navigation to Reduce Disparities in Cancer Care: Perspectives of Trained Navigators from the Field. J Canc Educ. 2011;26(1):111–20. 10.1007/s13187-010-0122-x. PMC3647146 20407860

[29] Sutter-Barrett R, Spencer NRB, Elnahas N, Hurd R, Delaney M, Bivens A. Navigating barriers and embracing facilitators of connection: insights from peer recovery specialists working with individuals with substance use disorder and criminal justice involvement: a qualitative analysis. Health Justice. 2025;13(1):7. 10.1186/s40352-024-00300-w. PMC11776266 39873840

[30] Matson TE, Navarro MA, Idu A, et al. Design of a cluster-randomized, hybrid type 1 effectiveness-implementation trial of a care navigation intervention to increase substance use disorder treatment engagement: study protocol. Addict Sci Clin Pract. 2025;20(1):78. 10.1186/s13722-025-00605-7. PMC12486859 41035041

[31] Green HM, Carmona-Barrera V, Diaz L, et al. Implementation of postpartum navigation for low-income individuals at an urban academic medical center. PLoS ONE. 2023;18(2):e0282048. 10.1371/journal.pone.0282048. PMC9949671 36821597

[32] Loo S, Mullikin K, Robbins C, et al. Patient navigator team perceptions on the implementation of a citywide breast cancer patient navigation protocol: a qualitative study. BMC Health Serv Res. 2022;22(1):683. 10.1186/s12913-022-08090-3. PMC9123866 35597947

[33] Tong A, Sainsbury P, Craig J. Consolidated criteria for reporting qualitative research (COREQ): a 32-item checklist for interviews and focus groups. Int J Qual Health Care. 2007;19(6):349–57. 10.1093/intqhc/mzm042. 17872937

[34] Palinkas LA, Horwitz SM, Green CA, Wisdom JP, Duan N, Hoagwood K. Purposeful Sampling for Qualitative Data Collection and Analysis in Mixed Method Implementation Research. Adm Policy Ment Health. 2015;42(5):533–44. 10.1007/s10488-013-0528-y. PMC4012002 24193818

[35] Hsu C, Mogk J, Hansell L, Glass JE, Allen C. Rapid Group Analysis Process (Rap-GAP): A Novel Approach to Expedite Qualitative Health Research Data Analysis. Int J Qualitative Methods. 2024;23:16094069241256275. 10.1177/16094069241256275. PMC12753008 41477558

[36] Harrison MS. Functions and Forms Framework: a Methodology for Mechanistic Deconstruction and Adaptation? J GEN INTERN MED. 2021;36(9):2809–11. 10.1007/s11606-020-06533-3. PMC8390705 33469751

[37] Perez Jolles M, Lengnick-Hall R, Mittman BS. Core Functions and Forms of Complex Health Interventions: a Patient-Centered Medical Home Illustration. J GEN INTERN MED. 2019;34(6):1032–8. 10.1007/s11606-018-4818-7. PMC6544719 30623387

[38] Spiro A, Oo SA, Marable D, Collins JP. A Unique Model of the Community Health Worker: The MGH Chelsea Community Health Improvement Team. Fam Community Health. 2012;35(2):147. 10.1097/FCH.0b013e3182465187. 22367262

[39] Valaitis RK, Carter N, Lam A, Nicholl J, Feather J, Cleghorn L. Implementation and maintenance of patient navigation programs linking primary care with community-based health and social services: a scoping literature review. BMC Health Serv Res. 2017;17(1):116. 10.1186/s12913-017-2046-1. PMC5294695 28166776

[40] Bernstein A, Merrilees J, Dulaney S, et al. Using care navigation to address caregiver burden in dementia: A qualitative case study analysis. Alzheimers Dement (N Y). 2020;6(1):e12010. 10.1002/trc2.12010. PMC7201177 32377557

[41] Kokorelias KM, Li Z, Hitzig SL. Understanding implementation characteristics in navigation programs for persons living with dementia and their caregivers: A scoping review. Int J Care Coord. 2023;26(2):62–74. 10.1177/20534345231151208.

[42] Chan RJ, Milch VE, Crawford-Williams F, et al. Patient navigation across the cancer care continuum: An overview of systematic reviews and emerging literature. Cancer J Clin. 2023;73(6):565–89. 10.3322/caac.21788. 37358040

[43] Center for substance abuse treatment. Integrating substance abuse treatment and vocational services. Substance Abuse and Mental Health Services Administration (US). 2000. http://www.ncbi.nlm.nih.gov/books/NBK64287/. Accessed October 23, 2025. 22514844

[44] Farhoudian A, Razaghi E, Hooshyari Z, et al. Barriers and Facilitators to Substance Use Disorder Treatment: An Overview of Systematic Reviews. Subst Abuse. 2022;16:11782218221118462. 10.1177/11782218221118462. PMC9434658 36062252

[45] Perron BE, Gillespie DF, Alexander-Eitzman B, Delva J. Availability of Outpatient Substance Use Disorder Treatment Programs in the United States. Subst Use Misuse. 2010;45(7–8):1097–111. 10.3109/10826080903443602. 20441453

