# Supplementary material for: Designing a patient-centered care navigation intervention within a healthcare system for SUD care: a qualitative study
Source: Addict Sci Clin Pract. 2026 Aug 14;21:86. doi: 10.1186/s13722-026-00712-z (PMC13757133; doi:10.1186/s13722-026-00712-z)
Supplement: Supplementary file 1 [file 13722_2026_712_MOESM3_ESM.pdf]

Addressing Barriers to Care for Substance Use Disorders
Patient Interview Guide

1. To start with, please tell me about your experience of talking to a care coordinator about getting connected with services for substance use.
   PROBE AS NEEDED:
   - [IF NEEDED, for ELABORATION] When I say, “care coordinator,” I’m referring to a licensed mental health worker who meets with patients one time, usually for 30-45 minutes by phone or video, to assess their mental health or substance use needs, determine a treatment plan, and make a referral for services. You may have talked to a care coordinator if you ever called KPWA asking for help with mental health or substance use.
   - [ELABORATION] How did you first get in touch with the care coordinator?
   - [ELABORATION] Why did you get in touch with a care coordinator?
     - [IF NOT FOR SUD] How did substance use come up?
   - [ELABORATION] What did you like about the process?
   - [ELABORATION] What did you dislike about the process?
   - [ELABORATION] How did you feel about the treatment plan that you and your care coordinator discussed?
   - [ELABORATION] What would you change about that treatment plan?

2. What got in the way of accessing substance use-related services after your call with a care coordinator?
   PROBE AS NEEDED:
   - [ELABORATION] What sort of logistical challenges did you face in accessing substance use services?
   - [ELABORATION] What sort of social or cultural factors made substance use services difficult or unappealing?
   - [ELABORATION] Can you tell me about any other personal challenges that you faced when accessing care?

3. Who or what helped you access substance use-related services?
   PROBE AS NEEDED:
   - [ELABORATION] What sort of practical or logistical factors helped you access care?
   - [ELABORATION] What sort of social or cultural factors made care appealing to you?
   - [ELABORATION] Can you tell me about any other personal factors that helped you access care?

We would like to offer patients extra support to help them access services for substance use. We are describing this extra support as “care navigation.” We plan on offering support to patients after their assessment appointment once a treatment plan is established. A care navigator, who is a licensed mental health clinician, will call patients within one week of their

assessment appointment to help them identify and resolve barriers to starting treatment. They will help the patient maintain motivation to engage in treatment and offer personalized support to potentially reduce disparities in access to treatment that might be based on language, income or geographical barriers, digital literacy, social and cultural factors, or other needs and preferences.

4. How would you feel about having someone reach out to you after your call with a care coordinator to help you follow-up on your treatment plan?
   PROBE AS NEEDED:
   - [ELABORATION] In what ways would it be helpful?
   - [ELABORATION] under what circumstances would it not be helpful?
   - [ELABORATION] What would make it most helpful?

5. Given your experience, what kinds of extra support would you like from a care navigator?
   PROBE AS NEEDED:
   - How would you like that help?
   - [ELABORATION] What resources could a care navigator provide that would be helpful to you?
   - [ELABORATION] What characteristics would you like to see in a care navigator?
   - [ELABORATION] How could a care navigator help you overcome barriers to accessing services for substance use-related care?

6. How would you like to learn about care navigation?
   PROBE AS NEEDED:
   - [ELABORATION] What would you want to know about care navigation before connecting with a care navigator?

7. How would you like to hear from someone who provides this extra support?
   PROBE AS NEEDED:
   - [ELABORATION] Would you prefer in-person, video, phone, text, or email communication? Or some other form of communication with the care navigator?
   - [ELABORATION] How soon after an assessment visit would you like to be in touch with a care navigator?
   - [ELABORATION] How often would you like a care navigator to contact you?

8. How would you like a care navigator to support your continued engagement in care?
   PROBE AS NEEDED:
   - [REPHRASED] Once you have accessed services for substance use, what factors impact your likelihood to stay engaged in care?

9. We are approaching the end of the interview. Now, I’d like you to imagine that you were seeking treatment and got help from a care navigator. Three weeks later, you felt that

the care navigator was helpful, and you were starting to accomplish your goals. What about care navigation would you say led to improvement?
PROBE AS NEEDED:
- [ELABORATION] How did that happen?

Thank you for sharing this information with me. Is there anything else you'd like to add before we end?
