# Supplementary material for: Designing a patient-centered care navigation intervention within a healthcare system for SUD care: a qualitative study
Source: Addict Sci Clin Pract. 2026 Aug 14;21:86. doi: 10.1186/s13722-026-00712-z (PMC13757133; doi:10.1186/s13722-026-00712-z)
Supplement: Supplementary file 2 [file 13722_2026_712_MOESM4_ESM.pdf]

Addressing Barriers to Care for Substance Use Disorders
Clinician Interview Guide

Patients who are seeking care for substance use may experience barriers and facilitators that affect their ability to start and continue treatment. We'd like to learn more about these barriers and facilitators, how we can design a care navigation intervention to help address them, and how to roll out this service in MHAC. So to start…

1. Consider the patients who you've referred to substance use treatment. What barriers do patients face when following through on referrals for substance use disorder treatment?
   PROBE AS NEEDED:
   - [ELABORATION] How might those barriers differ based on the type of treatment they are referred to? (For example: internal vs. external delivery systems)
   - [ELABORATION] How might those barriers differ for people of different racial groups, genders, sexual orientations, or socioeconomic status?

2. On the flip side, what factors help patients follow through on their referral for substance use treatment?

Now, I'd like your feedback on the project we are planning in collaboration with Kaiser Permanente Washington's Mental Health Access Center (MHAC). Our project assesses whether providing additional support to patients who are referred by a Mental Health Care Coordinator to substance use treatment increases their success in starting treatment. We plan on offering this support after their MHAC assessment appointment once a treatment plan has been established by the MHAC care coordinator.

This additional support comes in the form of a Care Navigator. The Care Navigator is a licensed mental health clinician who will call patients within one week of their assessment appointment to help them identify and resolve barriers to starting treatment. They may help motivate the patient to engage in treatment and offer support to address disparities experienced, which may be due to language, income or geographical barriers, digital literacy, or unmet needs or preferences

3. These next few questions are related to how we can best DESIGN the Care Navigator intervention …
   a. In an ideal world, what kind of extra support would you like the Care Navigator to provide to patients between when you talk to them and when they have their first treatment visit?
   b. How can a Care Navigator make it easier for KP Washington clinicians to treat or make referrals to treat patients with substance use?
   c. How can we design the Care Navigator role so that it is consistent with KP Washington's mission and current mental health and wellness efforts or initiatives?

d. What resources are available through KPWA that the Care Navigator could offer? What resources are not available through KPWA that you'd like the Care Navigator to provide?

Thank you for sharing your input on the design of a Care Navigation service. Now I want to ask you some questions about IMPLEMENTING Care Navigation into MHAC. We define "implementation" as all the processes and tasks that need to be done to start offering care navigation. This might include things that care coordinators, managers, patient access representative, and the entire MHAC department must develop and plan for. Examples might include attending trainings, creating job aids, and learning new Epic workflow. When implementation is successful, the process of offering Care Navigation is smoother.

4. What would be challenging or hard about IMPLEMENTING Care Navigation in MHAC?
   PROBE AS NEEDED:
   - [ELABORATION] Can you comment on any…
     - System-level challenges to implementation—things external to KPWA like policies or financing to pay for care navigation?
     - Organizational challenges to implementation—things like leadership, workflows, staffing capacity, and readiness to take on new initiatives?
     - Individual provider challenges to implementation—things like training needs, feeling overly burdened, or alignment with other practices?
     - Individual patient challenges to implementation—things like logistical or confidentiality concerns?

5. On the flip side, can you think of any strategies or things that would help us implement Care Navigation in MHAC?
   PROBE AS NEEDED:
   - [REPHRASED] What steps or adjustments would make it easier to integrate Care Navigation in MHAC?
   - [ELABORATION] Can you comment on any…
     - System-level strategies for implementation—things external to KPWA like policies, mandates, or community partnerships?
     - Organizational strategies for implementation—things like leadership, workflows, staffing capacity, and readiness to take on new initiatives?
     - Individual provider strategies for implementation—things like training, access to resources, or alignment with other practices?

6. Do you have concerns or doubts about this project? Can you envision that things will be easy, not a hassle, and pretty smooth about this project?

7. What are YOUR goals, your hopes, for participating in this project?
