# Supplementary material for: Designing a patient-centered care navigation intervention within a healthcare system for SUD care: a qualitative study
Source: Addict Sci Clin Pract. 2026 Aug 14;21:86. doi: 10.1186/s13722-026-00712-z (PMC13757133; doi:10.1186/s13722-026-00712-z)
Supplement: Supplementary file 3 [file 13722_2026_712_MOESM1_ESM.docx]

# APPENDIX A

## The Rap-GAP Method

The research team performed a novel rapid qualitative analysis method known as the Rapid Group Analysis Process (Rap-GAP) Method [(Hsu et al., 2024)](https://www.zotero.org/google-docs/?mOiAb3). The Rap-GAP method comprises five main steps to plan and conduct the rapid qualitative data analysis: (1) plan and prepare, (2) engage with data individually, (3) engage with data as a group, (4) collate learnings, and (5) summarize findings.

First, the Rap-GAP process leads (MN, TM, JG) identified three team members and two ABC-SUD interventionists to serve as Rap-GAP participants. The Rap-GAP leads co-designed the interview domains based on the interview guide, then generated an Excel spreadsheet template for each interview type with separate sheets for each interview domain. The Rap-GAP participants attended training led by the Rap-GAP leads on the rapid coding analysis. The Rap-GAP participants were then all assigned one patient transcript to use as training. After all participants extracted insights from the first transcript, participants met with the Rap-GAP leads to confirm the analysis was done correctly, then moved on to the next step.

Second, participants continued to log key insights and learnings on their own Excel spreadsheet. Insights were each listed under their respective domains and were accompanied by the respective de-identified quotes and the quotes’ interview IDs. Upon completion of the rapid coding analysis, a Rap-GAP lead compiled all insights and associated quotes onto one Excel spreadsheet. The Rap-GAP lead then calculated the number of quotes and Rap-GAP participants associated with each insight and noted which participant wrote each insight for quick reference. Insights were exported to Miro, a virtual whiteboard workspace, and grouped as Miro boards by interview type and domain. A Rap-GAP lead sent the Miro board to all Rap-GAP participants for individual review before the collaborative analysis in the next step.

Third, all Rap-GAP participants then attended one virtual meeting to gain training on the collaborative analysis and performed the group analysis of patient and clinician interview insights over a series of meetings. Meetings allowed a maximum of two participants to be absent in order to be held to ensure close adherence to the Rap-GAP method. During the group analysis, Rap-GAP participants split into teams of 2-3 and worked through each Miro board and aggregated similar insights into a common subtheme. Subthemes were then combined into common themes. After breakout teams completed their first round of data aggregation, all Rap-GAP participants regrouped to discuss additional questions and clarifications before reviewing the Miro boards together. Boards were then edited to reflect the larger group’s sentiments about subthemes and themes. Rap-GAP participants who were absent for any collaborative analysis meeting contributed their feedback to the aggregated boards discussed in that meeting asynchronously. A Rap-GAP lead, with the support of available Rap-GAP participants, generated bulleted summaries of key findings from each Miro board for the next step. Interview participants did not provide feedback on the findings from the Rap-GAP process; however relevant insights and illustrative quotes were shared with trial clinicians during training and high-level findings were presented to department leaders in an executive summary.

Fourth, the Rap-GAP leads met and organized the Rap-GAP summaries of key findings. Bulleted summaries were combined with their associated themes, subthemes, insights, and de-identified quotes with interview IDs in an Excel spreadsheet for quick reference during the final step. The results of the fifth step of the Rap-GAP process, the synthesis of insights into key themes, are reported in the main text.

## Figure A.1. Sample of Miro Virtual Whiteboard from Rap-GAP.
