# Supplementary material for: Designing a patient-centered care navigation intervention within a healthcare system for SUD care: a qualitative study
Source: Addict Sci Clin Pract. 2026 Aug 14;21:86. doi: 10.1186/s13722-026-00712-z (PMC13757133; doi:10.1186/s13722-026-00712-z)
Supplement: Supplementary file 4 [file 13722_2026_712_MOESM2_ESM.docx]

## APPENDIX B

## Figure B.1. Key Themes from Rap-GAP Method Related to SUD Care and care navigation Implementation across the Socio-ecological Framework.

The socioecological model has previously been used to organize contextual determinants of health and health behaviors. The model outlines levels of contextual factors to exist at differential proximity to the individual (i.e., community, organization, interpersonal, and individual level). This model helps define levels upon which different decisionmakers can intervene to improve health outcomes.

## **Table** B.**1. Frequency of Insights from Rap-GAP Method and Sample Quotes.**

Insights are organized using the socio-ecological model across interview domains. Sample quotes were omitted for insights that did not require further elaboration.

| Socio-ecological Model Level | Subtheme | Patients  (N, %) | Clinicians  (N, %) | Sample Quote |
| --- | --- | --- | --- | --- |
| Experiences with SUD Care | | | | |
| Pathways into care | Via medical resources | 9 (42.9) | - | "I ended up in the emergency department and then that stay at the emergency department just confirmed that I should be evaluated. I needed to take a close look at how I was using alcohol.” (PT #55) |
|  | Via self-motivation | 8 (38.1) | - | “I know that what I'm doing is damaging my health [and] I'm tired of being like this, of living like this. Mainly, I want to be healthy.” (PT #60) |
|  | Via health system resources | 5 (23.8) | - | "I called Kaiser and then they… forwarded me to it… Yes, member services, and they connected me.” (PT #22) |
|  | Via referral by counseling/social services | 5 (23.8) | - | “I spoke with a social worker, [and] she did an assessment and then I also expressed an interest about getting treatment at {CLINIC NAME} and she promptly got me a referral to them.” (PT #7) |
| Getting a treatment plan | Difficulties with follow-up | 3 (14.3) | - | “I couldn't get hold of [the clinician]. I tried the mental health department,… I tried to get customer service that there was a question that is not correctly entered… getting access to her or getting someone was totally impossible.” (PT #18) |
|  | Positive experience seeking treatment | 3 (14.3) | - | “It was a very positive experience. I felt like I was well heard and I felt like it guided me in a very appropriate manner.” (PT #55) |
|  | Patient was provided treatment options | 3 (14.3) | - | "[The clinician] gave me all these different treatment centers and I called them up.” (PT #18) |
| Experiences with treatment plan | Treatment option didn’t align with patient preferences | 2 (9.5) | - | “[I said] I would just like to know if you guys can put me on the medication that I need, because it helped me last time. [The clinician] just simply said no, you've got to go to treatment… It just felt so dismissive, that really hurt my feelings.” (PT #49) |
|  | Patient was provided a mental health treatment plan | 6 (28.6) | - | “They did… a mental health evaluation... [T]hey had somebody do another evaluation of me and then very quickly got me set up with an appointment for both mental health and substance abuse treatment the next day. It was actually very convenient.” (PT #22) |
| Themes Related to Seeking SUD Care | | | | |
| Community | Social stigma impacts linkage to SUD treatment. | 5 (23.8) | 1 (12.5) | "Interviewer: What sort of social or cultural factors might have made substance use services difficult or unappealing, if any? Participant: None, other than maybe the taboo nature of asking for help and I guess admitting to myself, you know.” (PT #1) |
|  | Social determinants of health impact linkage to SUD treatment. | 2 (9.5) | 6 (75.0) | “So if you were [born] into a middle upper class with financial stability with a great support system, family, member care, the work allows you to take time to go for your treatment and return to work…” (MHCC #3) |
| Organization | Navigating the continuum of care impacts linkage to SUD treatment. | 14 (66.7) | 7 (87.5) | “I think that’s why the care navigation is definitely needed. If we’re just saying okay go do this and then there’s no follow through I feel like it’s really easy to get nowhere. People are like uh, what was I supposed to do?” (MHCC #30) |
|  | Available and flexible treatment options impact linkage to SUD treatment. | 12 (57.1) | 7 (87.5) | “There’s times that they literally walk into the moment of weakness because obviously we know substance is really tempting everywhere. You know it’s easier to find substance than actually easier to find treatment facility.” (MHCC #3) |
|  | Misalignments of treatment and patient needs impact linkage to SUD treatment. | 4 (19.0) | 4 (50.0) | “...I think a biggest barrier is scheduling and time commitment. I think having groups or meetings during daytime hours when the majority of the population works, implies that our members have the ability to miss work or change their schedule, or things like that they have that privilege.” (MHCC #25) |
| Interpersonal | Effective communication with clinicians impacts linkage to SUD treatment. | 2 (9.5) | 2 (25.0) | “I felt like if there was somewhere to go and talk to the person in person, that will be better to know okay, that situation is not bad, and actually we can see you, you look fine, you're not shaking. Because I can tell you everything on the phone, but my appearance may be different.” (PT #42) |
|  | Support networks impact linkage to SUD treatment. | 13 (61.9) | 4 (50.0) | "My sister was in town. She’s a nurse and she has a history with behavioral health, and she was very kindly and gently encouraging me." (PT #55) |
| Individual | Patient’s motivation and readiness impacts linkage to SUD treatment. | 6 (28.6) | 5 (62.5) | “Sometimes people have a different change in their motivation and don’t follow through. I mean we can place a call to them to remind them. But it tends to make them the onus or take the responsibility of following through then their motivation may be wavering.” (MHCC #8) |
|  | Patient’s personal barriers to SUD care impact linkage to SUD treatment. | 2 (9.5) | 2 (25.0) | "Well, my level to manage stress, the way how I can make decisions [got in the way]. Sometimes you overthink it too much, when you go for help…” (PT #35) |
| Themes Related to Intervention Design | | | | |
| Organization | CNs should leverage SUD resources. | 17 (81.0) | 6 (85.0) | “Sometimes people need other resources outside of their mental health and substance use, like housing or job stuff because of their mental health and substance use. So yeah I think just getting more of like a team on board would be helpful.” (MHCC #30) |
|  | CNs should help patients navigate the continuum of care. | 12 (57.1) | 6 (75.0) | “Interviewer: Given your experience, what kinds of extra support would you like from a care navigator? Participant: Somebody to actually help find bed dates, a bed date to get into inpatient treatment, to help with getting into any kind of treatment, into outpatient or whatever. I guess them being with me to get those things set up.” (PT #30) |
|  | CNs should identify treatment options for patients. | 8 (38.1) | 4 (50.0) | “Maybe offering to help make appointments. It sounds like that’s what they’ll be doing anyway, but checking to make sure wherever you were referred was a good fit, if you’d rather try something else or somewhere else.” (PT #50) |
| Interpersonal | CNs should have critical positive traits. | 19 (90.5) | 6 (75.0) | "I think the idea of [care navigators] meeting people where they’re at. I mean just going from that lens, not having like… I understand we want people to show up to appointments, but if we’re too strict on those expectations, that’s going to make a lot of people stop going and attending services." (MHCC #25) |
|  | CNs should facilitate communication with care teams. | 17 (81.0) | 5 (62.5) | "Maybe what also would be helpful is to have them make sure things are coordinated between the primary care provider and the care service." (PT #50) |
|  | CNs should support patients throughout continuum of care.. | 9 (42.9) | 6 (75.0) | “Having a buddy sometimes could just be helpful, like whether it’s, I mean that’s I think why 12 step meetings work, it’s like having someone like walk with you through the process so that you’re not alone, is really useful.” (PT #30) |
| Individual | CNs should improve patient motivation. | 13 (61.9) | 2 (25.0) | “It’s just good to have constant reinforcement. I certainly wouldn’t want someone nagging me or making me feel bad if I don’t follow through right away, but having any follow-up is really good positive reinforcement that you want to do this, you want help, you want to work on it.” (PT #45) |
| Themes Related to Implementation | | | | |
| Community | Available community SUD resources facilitate implementation. | - | 5 (62.5) | “Yeah I think clinicians could be educated just on the resources that are available and then on… you know stages of motivation, that is often considered when interfacing with anybody that’s about to make changes. But then to apply that across the board.” (MHCC #8) |
|  | Community support for care navigation facilitates implementation. | - | 2 (25.0) | “...just finding out what other community supports are there and out there, because a lot of times it helps to [partner] with the community to provide these supports. It’s tough to know what’s what.” (MHCC #5) |
| Organization | Seamless integration of care navigation into existing workflows facilitates implementation. | - | 8 (100.0) | "The easiest way would be to add on the role of the care navigator to a current group. I feel like then there’s less to worry about with having too many people involved in workflows, and there’s a lot of workflows already kind of in place and MHAC did have a sort of teen navigator at some point which I think... some of this is similar to some of this is not." (MHCC #5) |
|  | Organizational support for care navigation facilitates implementation. | - | 6 (75.0) | “I mean you know in some ways it goes back to what I was saying about getting leadership buy in. They tell us they’re excited about something like a care navigator then by nature care coordinators will be excited about care navigator they can offer.” (MHCC #29) |
|  | Adequate staffing to support care navigation facilitates implementation. | - | 6 (75.0) | "Yeah it’s been tough to staff up at times. I think yeah finding staff and resources is always a problem with beginning that." (MHCC #5) |
|  | Training and psychoeducation focused on SUD facilitates implementation. | - | 2 (25.0) | "Yeah I think clinicians could be educated just on the resources that are available and then on… you know stages of motivation, that is often considered when interfacing with anybody that’s about to make changes." (MHCC #8) |

## Table B.2. Frequency of Insights from Rap-GAP Method Regarding Intervention Design.

Only the patient interview guide consisted of questions related to preferred methods and frequencies of contact prior to and during the care navigation intervention.

| Theme | Patients  (N, %) |
| --- | --- |
| Informing patients about care navigation | |
| Describe expectations of care navigation | 3 (14.3) |
| Describe role of CN, with respect to existing roles | 3 (14.3) |
| Timing of informing patients about care navigation | |
| Inform patient about care navigation promptly | 6 (28.6) |
| Method of informing patients about care navigation | |
| Via email | 8 (38.1) |
| Via text | 8 (38.1) |
| Via phone call | 7 (33.3) |
| Via online materials | 5 (23.8) |
| From care provider | 4 (19.0) |
| From clinician (i.e., MHCC) | 3 (14.3) |
| From someone at KPWA | 3 (14.3) |
| Via Kaiser app | 2 (9.5) |
| Via mail | 2 (9.5) |
| Frequency of communication with CN | |
| Within a few days of assessment | 13 (61.9) |
| Weekly | 7 (33.3) |
| As needed/ad hoc | 6 (28.6) |
| Biweekly | 4 (19.0) |
| Every few days | 4 (19.0) |
| Method of communication with CN | |
| Via phone call | 13 (61.9) |
| Via text | 8 (38.1) |
| Via email | 5 (23.8) |
| Via in-person | 3 (14.3) |
| Via video chat | 2 (9.5) |

CN: Care Navigator; KPWA: Kaiser Permanente Washington; MHCC: Clinician/KPWA mental health care coordinator
